# Accuracy of genotype imputation in Nelore cattle

**DOI:** 10.1186/s12711-014-0069-1

**Published:** 2014-10-10

**Authors:** Roberto Carvalheiro, Solomon A Boison, Haroldo H R Neves, Mehdi Sargolzaei, Flavio S Schenkel, Yuri T Utsunomiya, Ana Maria Pérez O’Brien, Johann Sölkner, John C McEwan, Curtis P Van Tassell, Tad S Sonstegard, José Fernando Garcia

**Affiliations:** UNESP, Universidade Estadual Paulista, Faculdade de Ciências Agrárias e Veterinárias, Jaboticabal, São Paulo 14884-900 Brazil; Division of Livestock Sciences, Department of Sustainable Agricultural Systems BOKU, University of Natural Resources and Life Sciences, Vienna, A-1180 Austria; GenSys Consultores Associados S/C Ltda, Porto Alegre, 90680-000 Brasil; Centre for Genetic Improvement of Livestock, University of Guelph, Guelph, ON N1G2W1 Canada; The Semex Alliance, Guelph, Ontario Canada; Centre for Reproduction and Genomics, AgResearch, Invermay, Mosgiel, New Zealand; United States Department of Agriculture, Agricultural Research Service, Bovine Functional Genomics Laboratory, Beltsville, MD 20705 USA; UNESP, Universidad Estadual Paulista, Faculdade de Medicina Veterinária de Araçatuba, Araçatuba, São Paulo 16050-680 Brazil

## Abstract

**Background:**

Genotype imputation from low-density (LD) to high-density single nucleotide polymorphism (SNP) chips is an important step before applying genomic selection, since denser chips tend to provide more reliable genomic predictions. Imputation methods rely partially on linkage disequilibrium between markers to infer unobserved genotypes. *Bos indicus* cattle (e.g. Nelore breed) are characterized, in general, by lower levels of linkage disequilibrium between genetic markers at short distances, compared to taurine breeds. Thus, it is important to evaluate the accuracy of imputation to better define which imputation method and chip are most appropriate for genomic applications in indicine breeds.

**Methods:**

Accuracy of genotype imputation in Nelore cattle was evaluated using different LD chips, imputation software and sets of animals. Twelve commercial and customized LD chips with densities ranging from 7 K to 75 K were tested. Customized LD chips were virtually designed taking into account minor allele frequency, linkage disequilibrium and distance between markers. Software programs FImpute and BEAGLE were applied to impute genotypes. From 995 bulls and 1247 cows that were genotyped with the Illumina® BovineHD chip (HD), 793 sires composed the reference set, and the remaining 202 younger sires and all the cows composed two separate validation sets for which genotypes were masked except for the SNPs of the LD chip that were to be tested.

**Results:**

Imputation accuracy increased with the SNP density of the LD chip. However, the gain in accuracy with LD chips with more than 15 K SNPs was relatively small because accuracy was already high at this density. Commercial and customized LD chips with equivalent densities presented similar results. FImpute outperformed BEAGLE for all LD chips and validation sets. Regardless of the imputation software used, accuracy tended to increase as the relatedness between imputed and reference animals increased, especially for the 7 K chip.

**Conclusions:**

If the Illumina® BovineHD is considered as the target chip for genomic applications in the Nelore breed, cost-effectiveness can be improved by genotyping part of the animals with a chip containing around 15 K useful SNPs and imputing their high-density missing genotypes with FImpute.

**Electronic supplementary material:**

The online version of this article (doi:10.1186/s12711-014-0069-1) contains supplementary material, which is available to authorized users.

## Background

Genomic information from dense single nucleotide polymorphism (SNP) chips provides the opportunity to increase the rate of genetic progress in breeding programs, if a sufficient number of markers and animals with phenotypes (or pseudo-phenotypes such as estimated breeding values, EBV) are genotyped [1]. Because the cost of genotyping is high, alternative methods are necessary for cost-efficient genomic applications. A strategy that is used in dairy breeding programs is to genotype influential animals using a denser chip (e.g. Illumina® BovineSNP50 v2 - 50 K; Illumina Inc., San Diego, CA) and selection candidates and cows using a lower-density chip (e.g. Illumina® BovineLD - 7 K) and then to impute (i.e. predict) missing genotypes from lower to higher density before calculating genomic estimated breeding values (GEBV) [2]. This cost-effective strategy provides reliabilities of GEBV that are similar to those obtained if selection candidates were genotyped with the higher-density chip [3,4].

The Nelore (indicine) breed is the most important beef cattle breed in Brazil [5]. For this breed, the Illumina® BovineHD chip (HD) is used as the “gold standard” for research purposes, since a low level of linkage disequilibrium between adjacent markers is observed in lower-density chips (e.g. 50 K) [6,7]. Profit margins from beef cattle operations are too low for the use of a HD chip at the commercial level. Thus, lower-density chips are required to overcome this limitation, which highlights the importance of assessing the accuracy of imputing genotypes in the Nelore breed.

The objective of this study was to assess the accuracy of genotype imputation in Nelore cattle, using different imputation methods, different commercial and customized SNP chips and sets of animals whose genotypes were to be imputed. The importance of relatedness between validation and reference animals was also evaluated for the different chips and methods.

## Methods

### Genotyped animals

Both sires and dams of the Nelore breed were genotyped. Sires that were widely used for artificial insemination were chosen as representative of the main Nelore breeding programs in Brazil. A total of 995 sires born between 1955 and 2008 were genotyped, spanning over 10 generations. A total of 1247 dams born between 1993 and 2008 were also genotyped. They included part of the genomic selection reference population of a commercial breeding program (DeltaGen) and were chosen among the dams that had the highest EBV accuracies for weaning, yearling and reproductive traits. Details about pedigree information of the genotyped animals are in Table 1.

**Table 1:** **Pedigree information of genotyped animals**

**Animals**	**Number**
Individuals in pedigree	9631
Sires	1536
Dams	6125
Individuals with progeny	7661
Individuals with no progeny	1970
Individuals with only known sire	17
Individuals with only known dam	1464
Individuals with known sire and dam	5067
Founders	3083
Founders with no progeny	350

To evaluate the accuracy of genotype imputation, the animals were divided into reference and validation sets. The reference set comprised 793 sires that were born before 2005. Two separate validation sets were considered, including the animals most likely to have their genotypes imputed in practice: the first with the 202 younger sires and the second with the dams. Reference sets with different numbers of animals were not considered because the effect of size of the reference set on imputation accuracy is well documented in the literature e.g. [3,8]. Statistics on genomic relationships between reference and validation sets are in Table 2. The genomic relationship matrix (**G**) was defined as **G** = **MM**′/Σ2p_i_(1-p_i_), in which **M** is the incidence matrix of markers whose elements in the i^th^ column are 0-2p_i_, 1-2p_i_ and 2-2p_i_ for genotypes AA, AB and BB, respectively, and p_i_ is the frequency of allele B at the i^th^ marker [9]. To compute **G**, only the HD markers that passed quality control (described later) were used. As shown in Table 2, young sires were, compared to dams, more related (on average) to the reference sires, based on the maximum (Maxr) or the average of the top 10 (Mean10) genomic relationships of a given animal in the validation set with all animals in the reference set.

**Table 2:** **Genomic relationship statistics between reference and validation sets**

**Reference/Validation sets** ^**1**^	**Statistic** ^**2**^	**Minimum**	**Maximum**	**Mean**	**Median**
Sire/sire (793; 202)	Maxr	0.0661	0.6241	0.4353	0.4677
	Mean10	0.0513	0.3795	0.2017	0.1970
Sire/dam (793; 1247)	Maxr	0.0392	0.6316	0.2813	0.2744
	Mean10	0.0333	0.3877	0.1351	0.1271

^1^Sire/sire: validation set composed of the 202 younger sires; sire/dam: validation set composed of 1247 dams; the same reference set of 793 sires was used in both cases; ^2^Maxr: maximum genomic relationship between each animal in the validation set and all the animals in the reference set; Mean10: average of the top 10 genomic relationships between each animal in the validation set and all the animals in the reference set.

### SNP chips

All sires and dams were genotyped with the HD chip, which contains approximately 777 K SNPs. Animals from the validation set had their HD genotypes masked, except for the genotypes of markers present on the LD chip under evaluation, thus mimicking a situation in which these animals were genotyped with LD chips.

Imputation from commercial LD panels to the HD chip was simulated by assuming that genotypes of animals from the validation set were available only for markers that were present on the HD and the following commercial chips: Illumina® BovineLD (7 K), Illumina® BovineSNP50 v2 (50 K) and GeneSeek® Genomic Profiler 20 K and 75 K for indicine breeds (GGP20Ki and GGP75Ki, respectively).

Eight customized (mimicked) 15 K LD chips were also tested, with varying densities and SNP selection criteria. Testing customized LD chips with less than 15 K SNPs was not relevant because their cost-effectiveness would not be attractive (Illumina®, personal communication). Using markers of the HD chip that passed quality control (see below), four 15 K chips were simulated based on selection of one marker from each window of 29 subsequent markers, according to the UMD v3.1 assembly. For the first 15 K chip (15 K_e), SNPs were evenly spaced by selecting the last marker from each window. For the second 15 K chip (15 K_em), the SNP with the highest minor allele frequency (MAF) was selected from each window, and for the third chip (15 K_el), the SNP with the highest average linkage disequilibrium with other SNPs from the same window was selected from each window. The r^2^ [10] was adopted as the measure of linkage disequilibrium. In the fourth 15 K chip (15K_eml), the SNP with the highest value for the product between its MAF and its average r^2^ with other SNPs from the same window was selected from each window.

The remaining four customized LD chips were developed based on the add-on concept offered by Illumina®, where additional SNPs can be added to an existing commercial chip in a cost-effective way. The Illumina® Bovine LD chip (7 K) was used as the base chip. Additional SNPs were selected using the same criteria as used for the 15K_eml chip. Windows containing 39, 25, 16 and 9 subsequent markers were used to compose chips with densities around 18 K (11a7 K), 24 K (17a7 K), 34 K (27a7 K) and 55 K (48a7 K) markers.

### Quality control of the genotypes

Quality control was performed for HD genotypes of the reference set, using the following criteria for excluding SNPs: (1) SNPs that were located in non-autosomal regions; (2) SNPs that had the same genomic coordinates, i.e. mapped to the same positions (just the replicates were removed); (3) SNPs with a p-value in the Hardy-Weinberg equilibrium z-test [11] less than or equal to 10^−5^; (4) SNPs with a MAF less than 0.02; and (5) SNPs with a call rate per SNP less than 0.98 (genotypes with a GenCall score less than 0.70 were considered missing when computing this statistic). After these edits, 439 595 SNPs remained. All samples from the reference set had a call rate per individual greater than 0.9 for SNPs passing quality control and were kept for the analyses.

SNPs excluded from the reference set were also discarded from the validation sets, in addition to masking subsets of HD SNPs as previously described. For each LD chip, the numbers of SNPs that were shared with the HD chip before and after quality control are in Table 3.

**Table 3:** **Number (Nb) of SNPs shared with the HD chip, for different SNP chips**

**SNP chip** ^1^	**Label**	**Nb common SNPs with HD**	**Nb common SNPs after QC** ^2^
Illumina® BovineHD	HD	777962	439595
Illumina® BovineLD	7 K	6637	4086
Illumina® Bovine SNP50 v2	50 K	49345	21014
GeneSeek® Genomic Profiler 20 K - Indicine	GGP20Ki	19493	13450
GeneSeek® Genomic Profiler 75 K - Indicine	GGP75Ki	73941	56169
Customized 15K_e	15K_e	15144	15144
Customized 15K_em	15K_em	15173	15173
Customized 15K_el	15K_el	15173	15173
Customized 15K_eml	15K_eml	15173	15173
Customized 11K_eml add-on 7 K	11a7 K	17841	15290
Customized 17 K_eml add-on 7 K	17a7 K	24121	21570
Customized 27 K_eml add-on 7 K	27a7 K	33942	31391
Customized 48K_eml add-on 7 K	48a7 K	55141	52590

^1^As described in the section “SNP chips” of “Methods”; ^2^QC: quality control of the genotypes.

### Imputation methods

Imputation of genotypes from the LD chips to the HD chip was performed using the software packages BEAGLE v.3.3 [12] and FImpute v.2.2 [13]. BEAGLE is a commonly used population-based imputation program (i.e. it does not rely on pedigree information) that adopts a stochastic procedure based on a Hidden Markov Monte-Carlo process to infer the probabilities of each haplotype/genotype. We used the most likely genotype as the predicted genotype. FImpute uses a family and population-based algorithm, or only the population-based algorithm, if pedigree information is not available, to deterministically phase the haplotypes and impute the missing genotypes. To evaluate the performance when considering family information in FImpute, we used both approaches, i.e. with or without pedigree information. Both programs were run with default parameters [12,13].

### Imputation scenarios

**Figure 1:**
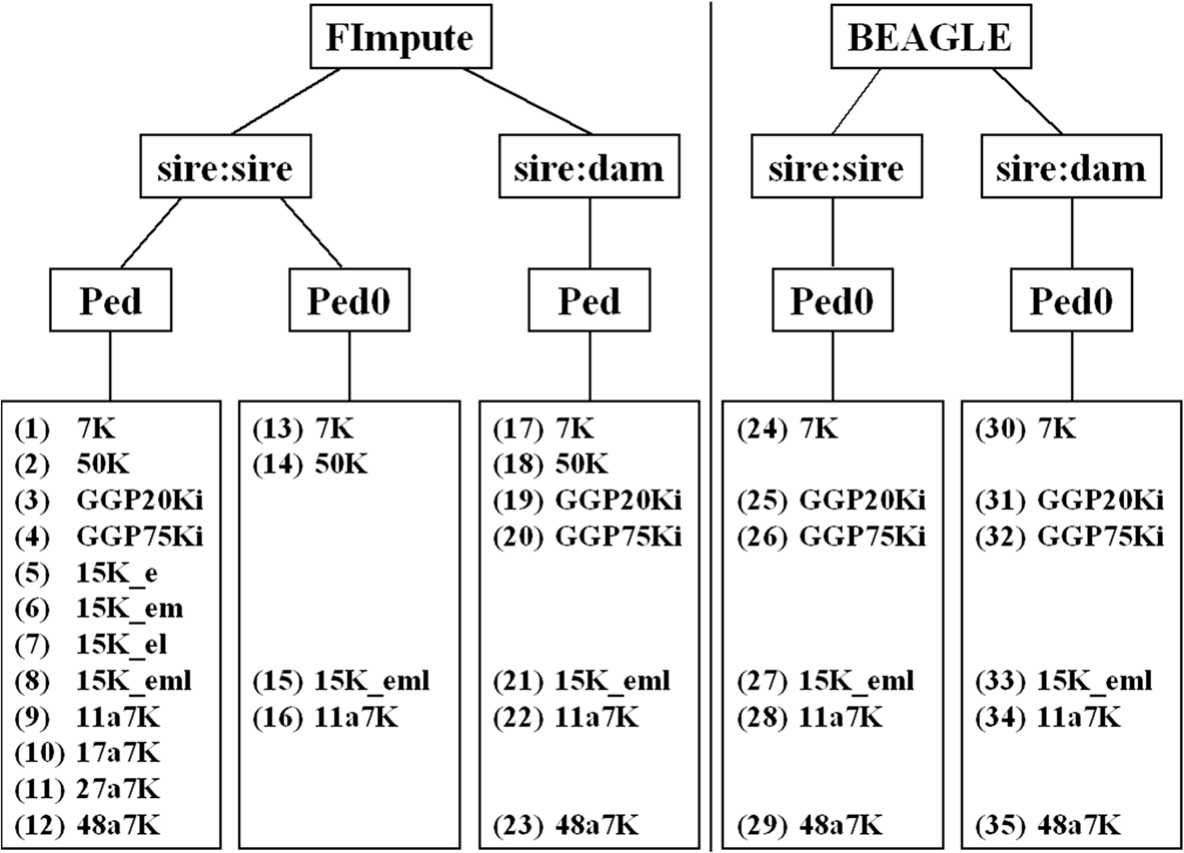
**Study design of the imputation analyses using FImpute and BEAGLE.** A reference set of 793 sires and a validation set with 202 young sires (sire:sire) or 1247 dams (sire:dam), with (Ped) or without (Ped0) pedigree information and different lower-density chips; numbers in brackets correspond to the number given to the analysis.

Considering the two sets of animals to be imputed (young sires and dams), the 12 SNP chips to be tested (7 K, 50 K, GGP20Ki, GGP75Ki, 15K_e, 15K_em, 15K_el, 15K_eml, 11a7 K, 17a7 K, 27a7 K, 48a7 K), and the three methods (BEAGLE and FImpute considering or ignoring pedigree), a complete factorial comparison would require 72 imputation analyses. As illustrated in Figure 1, only a subset of these analyses was carried out.

Analyses 1 to 12 were used to compare SNP chips. Results from analyses 13 to 16 were compared to those of their counterpart analyses (1, 2, 8 and 9, respectively) to evaluate the gain in accuracy when considering family information with FImpute. Results from analyses 17 to 23 were compared to those from analyses 1 to 4, 8, 9 and 12, to evaluate imputation accuracy when using different sets of animals to be imputed. Finally, analyses 24 to 29 and 30 to 35 were performed to infer accuracy of the imputed genotypes of Nelore young sires and dams using BEAGLE and different LD chips.

### Imputation accuracy

Two criteria were used to assess imputation accuracy. The first was the percentage of correctly imputed genotypes (PERC). For incorrectly imputed genotypes, either one or both alleles can be imputed incorrectly. To distinguish between these two cases, the Pearson’s correlation (CORR) between imputed and observed genotypes (coded as 0, 1 or 2 copies of the B allele) was also computed, as in Hickey et al. [14]. Both PERC and CORR were calculated by individual and by SNP, for imputed SNPs only. Since both BEAGLE and FImpute imputed all missing genotypes, statistics on the proportion of imputed SNPs were not needed.

The impact of genetic relatedness between validation and reference animals on imputation accuracy was assessed by regressing CORR on the average of the top 10 genomic relationships between each animal in the validation set with all the animals in the reference set [15].

## Results

### Comparison of lower-density SNP chips

Results for CORR and PERC obtained with the different LD chips (analyses 1 to 12) are in Table 4. Values for PERC were proportionally smaller than the corresponding values for CORR since the penalty for one incorrectly imputed allele is relatively higher for PERC than for CORR. Since both measures provided comparable results, imputation accuracy will be presented and discussed in terms of CORR.

**Table 4:** **Average (standard deviation) imputation accuracy, for different imputation analyses using FImpute**

**Analysis** ^**1**^	**SNP chip** ^**2**^	**Nb (%) SNPs to be imputed**	**CORR** ^**3**^	**PERC** ^**4**^
1	7 K	435509 (99.1)	0.9257 (0.0346)	90.56 (4.09)
2	50 K	418581 (95.2)	0.9783 (0.0136)	97.14 (1.76)
3	GGP20Ki	426145 (96.9)	0.9771 (0.0143)	96.96 (1.87)
4	GGP75Ki	383426 (87.2)	0.9922 (0.0056)	98.93 (0.76)
5	15K_e	424451 (96.6)	0.9784 (0.0135)	97.15 (1.75)
6	15K_em	424422 (96.5)	0.9820 (0.0120)	97.58 (1.61)
7	15K_el	424422 (96.5)	0.9763 (0.0138)	96.87 (1.77)
8	15K_eml	424422 (96.5)	0.9840 (0.0107)	97.85 (1.43)
9	11a7 K	424305 (96.5)	0.9823 (0.0117)	97.63 (1.54)
10	17a7 K	418025 (95.1)	0.9864 (0.0093)	98.17 (1.24)
11	27a7 K	408204 (92.9)	0.9897 (0.0072)	98.60 (0.97)
12	48a7 K	387005 (88.0)	0.9931 (0.0049)	99.05 (0.67)

^1^Imputation analyses using FImpute (considering family information) and 202 young sires as the validation set; the numbers of each analysis refer to those in brackets from Figure 1; ^2^as described in the section “SNP chips” of “Methods”; ^3^CORR: Pearson’s correlation between imputed and observed genotypes; ^4^PERC: percentage of correctly imputed genotypes.

Imputation accuracy was greater than 0.97 for all chips except for the 7 K chip. Considering the proportion of SNPs to be imputed (99.1%), the imputation accuracy was high even for the 7 K chip. As documented in the literature [4,16], imputation accuracy increases with a decreasing proportion of SNPs to be imputed. However, the gain in accuracy from the 11a7 K to the 48a7 K chip, for example, was small (0.0108) because the accuracy obtained with the 11a7 K chip was already high.

Among the commercial chips, imputation accuracies with the 50 K and GGP20Ki chips were similar and outperformed that of the 7 K chip, while the GGP75Ki chip had the best accuracy. Among the virtual 15 K chips, selecting SNPs based on MAF (15K_em) rather than on linkage disequilibrium (15K_el), in addition of being evenly spaced, tended to result in slightly higher imputation accuracies. The highest accuracy was observed when both criteria and even spacing were combined to define the SNP content of a virtual chip (15K_eml). However, the increase in accuracy was nominal compared to the 15K_e evenly spaced chip. Imputation accuracy of the 11a7 K chip was comparable to that of 15K_eml, with the potential benefit of the former being cheaper to be manufactured/acquired by adding SNPs on the existing 7 K chip.

Although commercial and customized chips resulted in similar imputation accuracies, the customized chips that had the highest accuracies outperformed commercial chips with a similar density (after quality control). For instance, the average accuracy of the 15K_eml chip was 0.6% and 0.7% higher than those of the 50 K and GGP20Ki chips, respectively.

### Importance of pedigree information

Results of FImpute analyses with and without pedigree information are in Table 5. For the 50 K, 15K_eml and 11a7 K chips, there was no benefit from using pedigree information and for the 7 K chip, the gain in accuracy was marginal (+1%). These low gains in accuracy are in part due to the low quality of the available pedigree information, since the sire was unknown for a proportion (38%) of the genotyped animals from the validation set, but also because FImpute assumes that all animals are to some degree related when performing population imputation, by searching for common haplotypes shared by individuals [13].

**Table 5:** **Average (standard deviation) imputation accuracy, using FImpute with or without pedigree (Ped) information**

**Analyses** ^**1**^	**SNP chip** ^**2**^	**With Ped**	**Without Ped**
1 and 13	7 K	0.9257 (0.0346)	0.9164 (0.0351)
2 and 14	50 K	0.9783 (0.0136)	0.9781 (0.0132)
8 and 15	15K_eml	0.9840 (0.0107)	0.9832 (0.0113)
9 and 16	11a7 K	0.9823 (0.0117)	0.9819 (0.0120)

^1^Imputation analyses using FImpute software and 202 younger sires as the validation set; the numbers of each analysis refer to those in brackets from Figure 1; the first and the second numbers refer to analyses with and without pedigree information, respectively; ^2^as described in the section “SNP chips” of “Methods”.

### Comparison of validation sets

Imputation accuracy was lower for dams than for young sires, especially for lower density chips (Table 6). For instance, the difference in accuracy between young sires and dams was 5.0% for the 7 K chip and 0.7% for the 48a7 K chip. As a consequence, the increase in accuracy for dams was more pronounced as the proportion of SNPs to be imputed decreased. This result is due to the fact that the young sires were, on average, more related to the reference sires than the dams (Table 2). This effect of relatedness on imputation accuracy will be discussed in more detail below.

**Table 6:** **Average (standard deviation) imputation accuracy, using dams or young sires as validation set**

**Analyses** ^**1**^	**SNP chip** ^**2**^	**Dams**	**Young sires**
17 and 1	7 K	0.8791 (0.0474)	0.9257 (0.0346)
18 and 2	50 K	0.9603 (0.0190)	0.9783 (0.0136)
19 and 3	GGP20Ki	0.9566 (0.0211)	0.9771 (0.0143)
20 and 4	GGP75Ki	0.9846 (0.0082)	0.9922 (0.0056)
21 and 8	15K_eml	0.9680 (0.0164)	0.9840 (0.0107)
22 and 9	11a7 K	0.9658 (0.0173)	0.9823 (0.0117)
23 and 12	48a7 K	0.9864 (0.0070)	0.9931 (0.0049)

^1^Imputation analyses using FImpute (considering family information) and different validation sets; the numbers of each analysis refer to those in brackets from Figure 1; the first and the second numbers refer to analyses using dams or young sires as validation set, respectively; ^2^as described in the section “SNP chips” of “Methods”.

### Comparison of imputation methods

FImpute outperformed BEAGLE for the different chips and validation sets in terms of average accuracy (Table 7). The greatest difference was observed for the validation set of dams and the 7 K chip, for which the average accuracy of FImpute was 3.4% higher than that of BEAGLE. The average differences in accuracy between FImpute and BEAGLE were more pronounced for dams (1.5 to 3.4%) than for young sires (0.7 to 3.1%). For both validation sets, differences between imputation methods tended to be higher at lower densities. BEAGLE also presented, for all chips, minimum accuracy values that were lower than those of FImpute. The minimum accuracies obtained with BEAGLE were on average 4.6% and 4.7% lower for young sires and dams, respectively, than the minimum accuracies for the corresponding FImpute analyses.

**Table 7:** **Summary statistics of imputation accuracy, using BEAGLE and FImpute**

			**BEAGLE (FImpute)**			
**Anal.** ^**1**^	**Validation set**	**SNP chip** ^**2**^	**Minimum**	**Maximum**	**Mean**	**SD**
24 (1)	Young sire	7 K	0.7525 (0.8003)	0.9717 (0.9845)	0.8982 (0.9257)	0.0392 (0.0346)
25 (3)	Young sire	GGP20Ki	0.8603 (0.8988)	0.9951 (0.9963)	0.9614 (0.9771)	0.0225 (0.0143)
26 (4)	Young sire	GGP75Ki	0.9142 (0.9568)	0.9986 (0.9990)	0.9842 (0.9922)	0.0120 (0.0056)
27 (8)	Young sire	15K_eml	0.8788 (0.9211)	0.9976 (0.9981)	0.9714 (0.9840)	0.0183 (0.0107)
28 (9)	Young sire	11a7 K	0.8773 (0.9163)	0.9979 (0.9975)	0.9697 (0.9823)	0.0190 (0.0117)
29 (12)	Young sire	48a7 K	0.9214 (0.9628)	0.9989 (0.9992)	0.9860 (0.9931)	0.0111 (0.0049)
30 (17)	Dam	7 K	0.6969 (0.7096)	0.9576 (0.9656)	0.8501 (0.8791)	0.0441 (0.0474)
31 (19)	Dam	GGP20Ki	0.8124 (0.8357)	0.9874 (0.9923)	0.9321 (0.9566)	0.0288 (0.0211)
32 (20)	Dam	GGP75Ki	0.8645 (0.9291)	0.9946 (0.9976)	0.9692 (0.9846)	0.0198 (0.0082)
33 (21)	Dam	15K_eml	0.8296 (0.8711)	0.9904 (0.9954)	0.9456 (0.9680)	0.0254 (0.0164)
34 (22)	Dam	11a7K	0.8249 (0.8640)	0.9893 (0.9951)	0.9430 (0.9658)	0.0260 (0.0173)
35 (23)	Dam	48a7K	0.8677 (0.9363)	0.9954 (0.9980)	0.9715 (0.9864)	0.0193 (0.0073)

^1^Results of imputation analyses using BEAGLE or FImpute (between brackets) and different validation sets (young sires and dams); the numbers of each analysis refer to those from Figure 1; ^2^as described in the section “SNP chips” of “Methods”; SD = standard deviation.

### Importance of genomic relatedness for imputation accuracy

**Figure 2:**
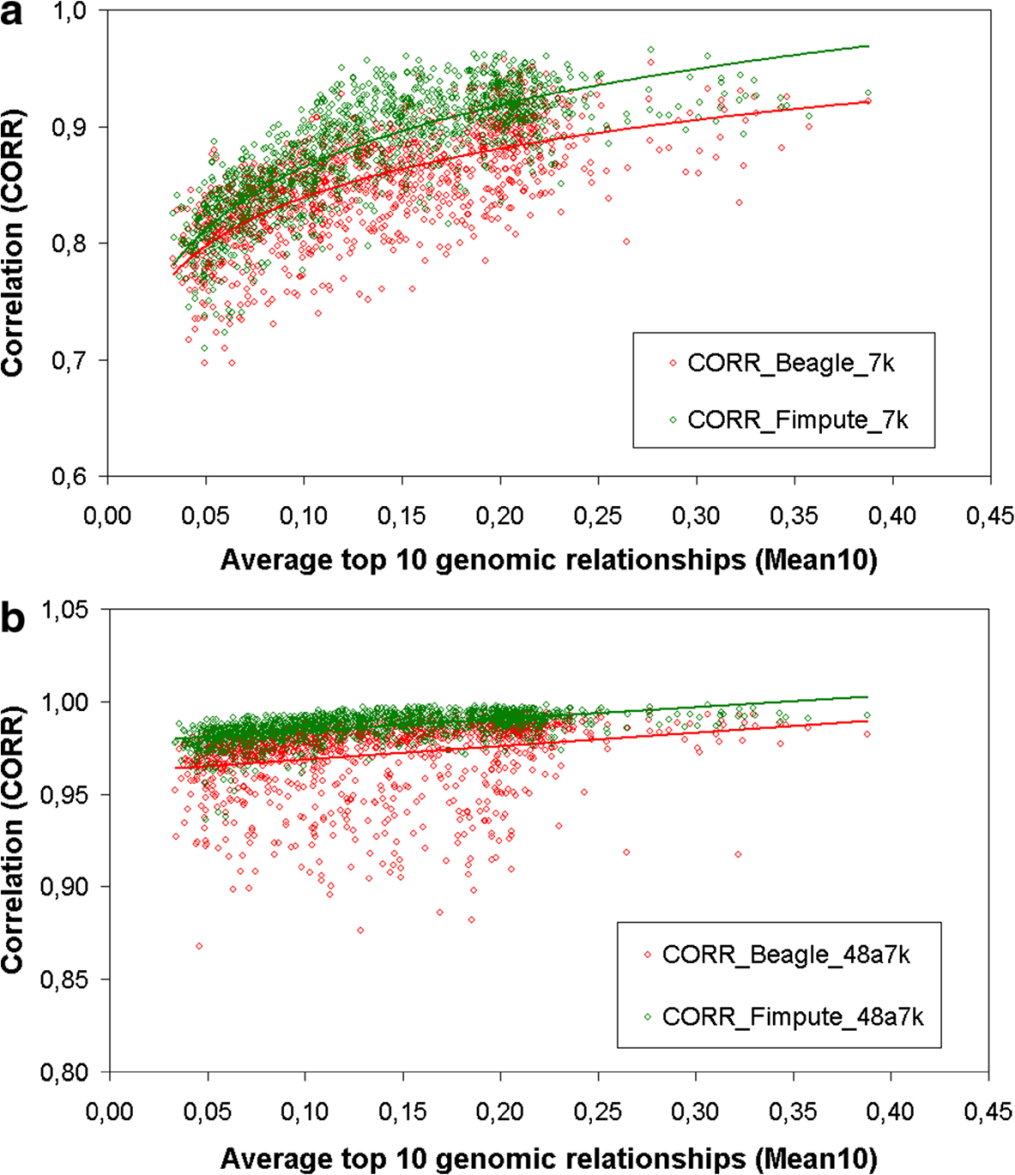
**Accuracy of imputation (CORR) as a function of genomic relatedness (Mean10), using BEAGLE and FImpute.** Figure 2 shows the results from the imputation analyses using dams as the validation set and the 7 K (top) or 48a7 K (bottom) chip. Solid lines refer to second order polynomial (top) and linear (bottom) regressions.

The impact of relatedness between validation and reference animals on imputation accuracy is illustrated in Figure 2. For the purpose of clarity, only results of four representative analyses (17 and 30; 23 and 35) are presented. The impact of relatedness to the reference set on accuracy was more evident for lower (7 K) than for higher density (48a7 K) chips. Imputation accuracies tended to be higher as the relatedness between imputed and reference animals increased. This tendency was stronger for FImpute than for BEAGLE with the 7 K chip and was similar, on average, between both software in the 48a7 K chip. For below average levels of relatedness, the dispersion of imputation accuracies was higher for BEAGLE than for FImpute, notably for the 48a7 K chip.

### SNP-wise imputation accuracy

**Figure 3:**
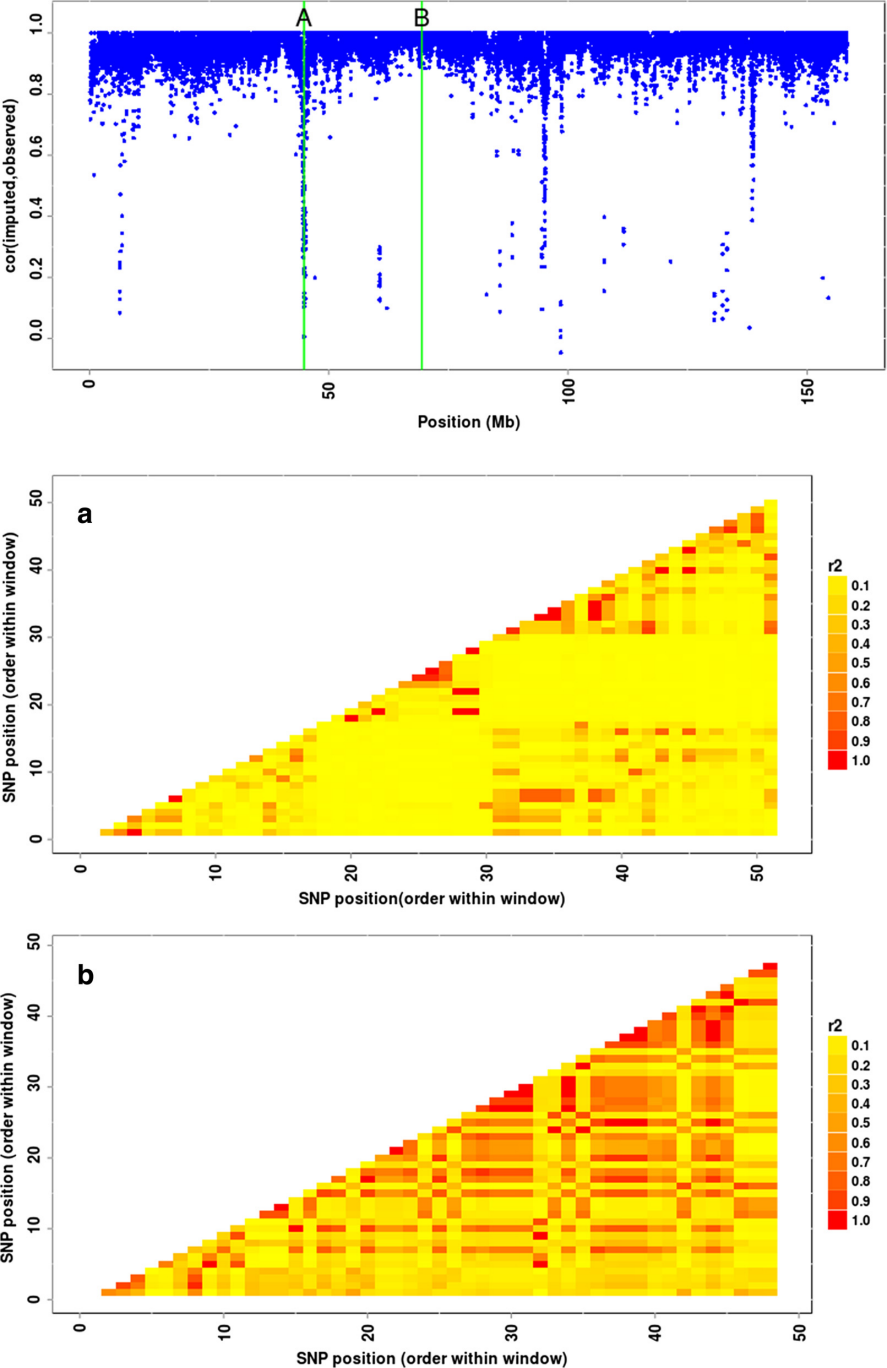
Variation of SNP-wise imputation accuracy* and linkage disequilibrium along bovine chromosome 1. Top: SNP-wise correlation between imputed and observed genotypes (CORR) is plotted against the genomic coordinates (in Mb) for SNPs located on chromosome 1, which was divided in windows of about 50 subsequent markers; windows with the lowest **(a)** and highest **(b)** average imputation accuracies are highlighted. Middle: Heatmap representing the extent of linkage disequilibrium (r^2^) in window A (51 markers located between 44.71 and 44.91 Mb; averages for accuracy, MAF and r^2^ were 0.390, 0.195 and 0.103, respectively). Bottom: Heatmap representing the extent of r^2^ in window B (48 markers located between 69.40 and 69.49 Mb; averages for accuracy, MAF and r^2^ were 1.000, 0.270 and 0.321, respectively). *In order to exemplify the amount of variation verified for SNP-wise imputation accuracy on a single chromosome, the results obtained from Analysis 9 (Figure 1) are presented (i.e. using the 11a7k chip and FImpute considering pedigree information to impute genotypes of young sires).

Although imputation accuracy was in general high, SNP-wise imputation accuracy is relevant also. For brevity, only the result of analysis 9 for bovine autosome 1 is presented in Figure 3. As previously reported in the literature for other cattle breeds [17,18], some regions of the genome had very low imputation accuracy (CORR < 0.60). A more careful analysis revealed that these regions had markers with very low levels of linkage disequilibrium with neighboring markers (Figure 3), which suggests potential mapping or assembly issues in the reference genome. Comparatively, BEAGLE and FImpute had low imputation accuracy for the same genomic regions (data not shown). Markers of these regions were removed in an attempt to increase imputation accuracy of the markers from the surrounding regions, but no improvements on imputation accuracy were obtained, possibly because the proportion of discarded markers was small and the imputation accuracies were already high in the neighboring regions (data not shown).

## Discussion

Imputation methods rely partially on linkage disequilibrium between markers to infer unobserved genotypes. The Nelore breed has lower levels of linkage disequilibrium at short distances than taurine breeds [6,7]. Nevertheless, the imputation accuracies obtained in this study are comparable to accuracies reported in the literature for taurine breeds [17-19]. For instance, Ma et al. [19] imputed genotypes from the 50 K to the HD chip in a population of Swedish and Finnish Red cattle and found similar accuracies (around 0.97) to those reported here. As documented in the literature e.g. [3,8,16], imputation accuracy increased with increasing density of the LD chip. Increasing the density of the LD chip from 7 K to 15 K resulted in a greater increase in imputation accuracy than an increase in density from 15 K to 75 K, because imputation accuracy was already high (>0.97) for the 15 K chip. This result is consistent, for example, with those of Khatkar et al. [3] who reported on the imputation of 50 K genotypes of Australian Holstein-Friesian cattle and observed a relatively greater increase in imputation accuracy when the density of the LD chip increased from 3 K to 7 K than from 7 K to 10 K.

Imputation accuracy has a large influence on the reliability of genomic predictions [3,4,20]. Mulder et al. [4] derived a deterministic equation to predict the accuracy of GEBV based on imputation accuracy (measured as a correlation) and observed that it increased linearly with increasing imputation correlation. Daetwyler et al. [21] suggested that the decline in accuracy of GEBV was actually slightly lower than the decline in accuracy of imputation. In a scenario with low-density genotypes (14 SNPs/Morgan), these authors observed that 87.8% of missing genotypes were correctly imputed but 95% of the accuracy of GEBV obtained with high-density SNP genotypes (1500 SNPs/Morgan) was achieved. Although reliability of GEBV was not evaluated in the present study, it is plausible to assume that the reliability of GEBV of Nelore cattle based on imputed genotypes from a chip with approximately 15 K SNPs, for which the imputation accuracy was around 0.98, would be similar to that of GEBV obtained with the HD chip. Nevertheless, a more thorough analysis on this subject is needed.

Different (mimicked) customized LD chips were tested in order to evaluate changes in imputation accuracy when criteria for SNP selection were modified. As in Mulder et al. [4], selecting SNPs based on MAF (15K_em) and even spacing across the genome had little impact on imputation accuracy compared to selecting SNPs based only on even spacing. A small favorable difference was observed when SNPs were selected based on MAF (15K_em) rather than on linkage disequilibrium (15K_el), in addition to being evenly spaced. A slightly better accuracy was observed when both criteria (MAF and linkage disequilibrium), in addition to even spacing, were combined to select the SNPs for the chip (15K_eml). Increasing SNP density in the telomere regions of the chromosomes is expected to further increase the imputation performance of the customized chips [22]. We did not use refined algorithms to optimize the imputation accuracy of the customized chips since the development of an LD chip was outside the scope of our study. Results obtained with the 11a7 K chip suggests that if a new optimized LD chip was to be developed, adding SNPs to the existing commercial 7 K chip would be a good strategy since this would be less costly and provide an imputation accuracy that is comparable to that of a completely customized chip with similar density.

The customized chips that showed the highest imputation accuracy slightly outperformed the commercial chips with an equivalent density. It is important to mention that commercial and customized chips cannot be properly compared, since the design of the customized chips used information on genotypes from the same population than that to be imputed. However, it does highlight the importance of using population-specific information to design LD chips.

Imputation accuracy was not as much affected by pedigree information as by using different imputation methods. FImpute resulted in higher imputation accuracies for the different chips and validation sets than BEAGLE. Ma et al. [19] found that FImpute slightly outperformed BEAGLE when imputing Swedish and Finnish Red cattle genotypes from 50 K to HD, but BEAGLE outperformed FImpute when imputing from 3 K to 50 K. Sun et al. [23] also observed a slightly better imputation performance of BEAGLE compared to FImpute when imputing Angus genotypes from 7 K to 50 K. These results indicate that the choice of the imputation method depends on the chip and population, i.e. there is no single method that provides higher imputation accuracy for all scenarios. However, an outstanding advantage of FImpute over BEAGLE is its computational efficiency. As reported by Ma et al. [19], processing time of the analysis with FImpute was much shorter than with BEAGLE (data not shown).

Another factor that influenced imputation accuracy was the level of relatedness between imputed and reference animals. In agreement with the literature [14,19,24,25], imputation accuracy tended to increase as the relatedness between imputed and reference animals increased for both imputation methods. The influence of relatedness on imputation accuracy decreased with increasing SNP density of the LD chip. For the Nelore cattle population, using the denser LD chips (GGP75Ki and 48a7 K) resulted in high accuracies (>0.90) with FImpute even for animals that were poorly related to the reference set.

For some genomic applications (e.g. genome-wide association (GWA) studies), SNP-wise imputation accuracy is relevant to prevent the propagation of genotyping errors. As in Erbe et al. [17] and VanRaden et al. [18], some regions of the genome contained markers that presented an erratic pattern of linkage disequilibrium, which suggests potential mapping and reference genome assembly problems. These regions had a negligible effect on imputation accuracy by individual but can potentially affect GWA studies. While the origin of this erratic linkage disequilibrium pattern is unknown, a precautious strategy would be to exclude markers from these regions as a quality control criterion for GWA studies. To facilitate this, a supplementary table [See Additional file 1: Table S1] summarizes all the observed regions that presented poor imputation performance.

Finally, it is not clear if the Illumina® BovineHD chip should be considered as the target high-density chip for genomic applications in the Nelore breed. Recent genomic prediction results (not published) have revealed that the 50 K and HD chips share similar predictive abilities for different traits in Nelore cattle. At present, it is not clear to what extent the size and composition of the reference population influence these results. As reported by VanRaden et al. [20], the benefit of using denser chips for genomic prediction becomes more evident as the reference population increases. Denser chips are also preferred for genomic applications that aim at identifying and subsequently using information from causal mutations [26,27]. Except for the most valuable breeding stock (e.g. influential sires and potential donor cows), genotyping the animals with dense chips is prohibitive for most beef cattle operations. Thus, genotyping strategies need to be further investigated to allow the incorporation of genomic information in beef cattle breeding programs in a cost-effective way. The results presented here show that a strategy of genotyping dams and young sire candidates with LD chips to predict missing HD genotypes by imputation is feasible. Future studies are needed to better identify the proper densities of genotyping chips to be used for each category of animals and in which proportion they should be genotyped for each application.

## Conclusions

Our results indicate that if the HD chip is considered as the target chip for genomic applications in the Nelore breed, cost-effectiveness can be improved by genotyping part of the economically marginal animals with an LD chip that contains around 15 K useful SNPs and imputing the missing HD genotypes. A denser LD chip (50 K useful SNPs) is recommended for animals that are poorly related to the reference population. For the current Nelore population, FImpute is preferred over BEAGLE for imputation of missing genotypes.

## Additional file

Additional file 1:
**Observed autosome regions presenting an average imputation accuracy (CORR) lower than 0.7.** BTA refers to the bovine (*Bos taurus*) autosome for which the window was mapped; min.win is the position in base pairs (according to Illumina® map) of the first SNP of the window; max.win is the position in base pairs (according to Illumina® map) of the last SNP of the window; CORR(sdCORR) is the average (SD) of the correlation between observed and imputed genotypes, considering the SNP within the window; PERC(sdPERC) is the average (SD) of the percentage of correctly imputed genotypes, considering the SNP within the window; mMAF(sMAF) is the average (SD) of MAF, considering the SNP within the window; mr2(sr2) is the average (SD) of LD (r^2^) between all pairs of SNPs located in the same window; medr2 is the median of LD (r^2^) between all pairs of SNP located in the same window; win is the window index (consecutive numbers on the same chromosome indicate contiguous windows); Nsnp is the number of markers in the window; avg_dist is the average gap between adjacent markers within the same window (in kb).
